# Supplementary material for: Identification and Characterization of MicroRNAs in Gonads of Helicoverpa armigera (Lepidoptera: Noctuidae)
Source: Insects. 2021 Aug 19;12(8):749. doi: 10.3390/insects12080749 (PMC8396854; doi:10.3390/insects12080749)
Supplement: Supplementary file 1 [file insects-12-00749-s001.zip › Supplementary Files/Table S1.pdf]

Table S1. Stem-loop RT-PCR and qPCR primers of miRNAs of *H. armigera*.

miRNA ID	qPCR primer(5′-3′)	Stem-loop RT primer(5′-3′)
miR-252a-5p	F:CTAAGTACTAGTGCCGCAGGAG<br>R:GTGCAGGGTCCGAGGTATTC	GTCGTATCCAGTGCAGGGTCCGAGGTA<br>TTCGCACTGGATACGACCTCCTG
miR-34	F:TGGCAGTGTGGTTAGCTGGTT<br>R:GTGCAGGGTCCGAGGTATTC	GTCGTATCCAGTGCAGGGTCCGAGGTA<br>TTCGCACTGGATACGACAACCAG
miR-989a	F:GTGTGATGTGACGTAGTGGAAG<br>R:GTGCAGGGTCCGAGGTATTC	GTCGTATCCAGTGCAGGGTCCGAGGTA<br>TTCGCACTGGATACGACCTTCCA
miR-1a-5p	F:TGGAATGTAAAGAAGTATGGAG<br>R:GTGCAGGGTCCGAGGTATTC	GTCGTATCCAGTGCAGGGTCCGAGGTA<br>TTCGCACTGGATACGACCTCCAT
miR-263a-5p	F:AATGGCACTGGAAGAATTCACGGG<br>R:GTGCAGGGTCCGAGGTATTC	GTCGTATCCAGTGCAGGGTCCGAGGTA<br>TTCGCACTGGATACGACCCCGTG
miR-31-5p	F: AGGCAAGAAGTCGGCATAGCTGT<br>R:GTGCAGGGTCCGAGGTATTC	GTCGTATCCAGTGCAGGGTCCGAGGTA<br>TTCGCACTGGATACGACACAGCT
miR-2c	F: TATCACAGCCAGCTTTGTTGACT<br>R:GTGCAGGGTCCGAGGTATTC	GTCGTATCCAGTGCAGGGTCCGAGGTA<br>TTCGCACTGGATACGACAGTCAA
miR-2763	F: ATATTATGCTCATTACTTTGGAT<br>R:GTGCAGGGTCCGAGGTATTC	GTCGTATCCAGTGCAGGGTCCGAGGTA<br>TTCGCACTGGATACGACATCCAA
miR-998	F: TAGCACCATGGGATTCAGCTC<br>R:GTGCAGGGTCCGAGGTATTC	GTCGTATCCAGTGCAGGGTCCGAGGTA<br>TTCGCACTGGATACGACGAGCTG
miR-2765	F: TGGTAACTCCACCACCGTTGGC<br>R:GTGCAGGGTCCGAGGTATTC	GTCGTATCCAGTGCAGGGTCCGAGGTA<br>TTCGCACTGGATACGACGCCAAC
miR-263b-5p	F: CTTGGCACTGGGAGAATTCAC<br>R:GTGCAGGGTCCGAGGTATTC	GTCGTATCCAGTGCAGGGTCCGAGGTA<br>TTCGCACTGGATACGACGTGCCT
U6	F: AGGATGACACGCAAAATCGT<br>R:GTGCAGGGTCCGAGGTATTC	GTCGTATCCAGTGCAGGGTCCGAGGTA<br>TTCGCACTGGATACGACACGATT
let-7	F: GCGGCGGTAGTAGGTTGTAT<br>R:GTGCAGGGTCCGAGGTATTC	GTCGTATCCAGTGCAGGGTCCGAGGTA<br>TTCGCACTGGATACGACATACAA
