# Supplementary figures and images for: Identification and Characterization of MicroRNAs in Gonads of Helicoverpa armigera (Lepidoptera: Noctuidae)
Source: Insects. 2021 Aug 19;12(8):749. doi: 10.3390/insects12080749 (PMC8396854; doi:10.3390/insects12080749)

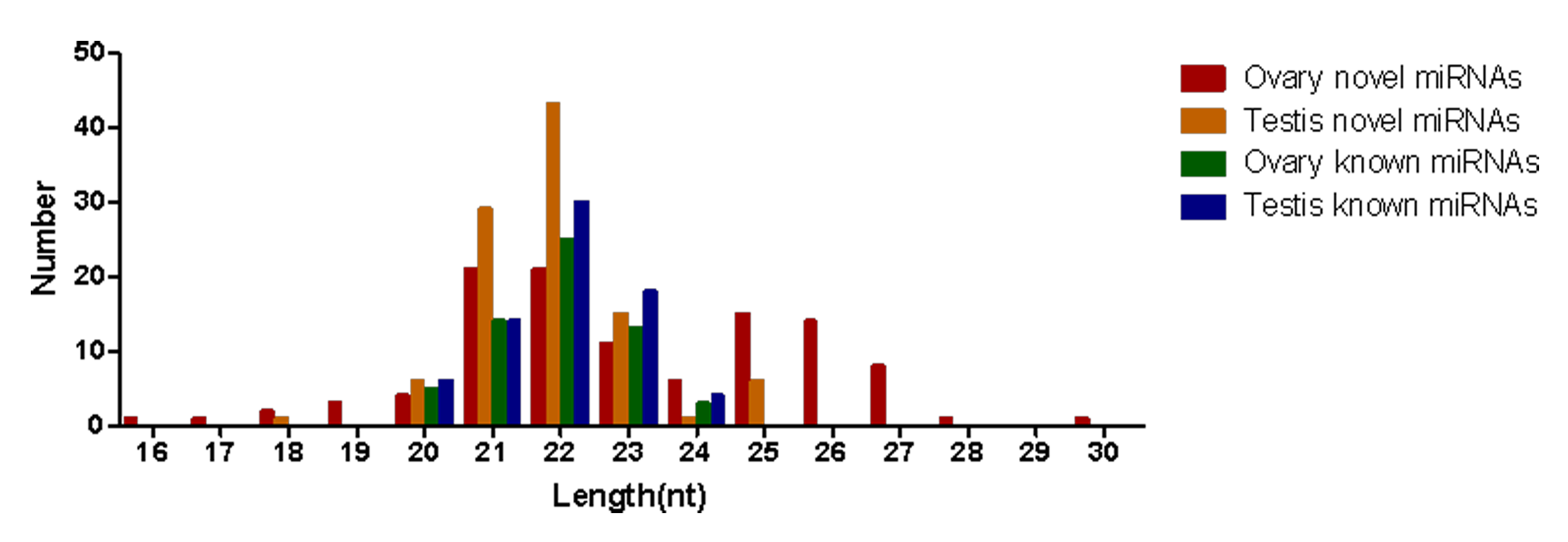

Supplement: Supplementary file 1 [file insects-12-00749-s001.zip › Supplementary Files/Figure S1.tif]

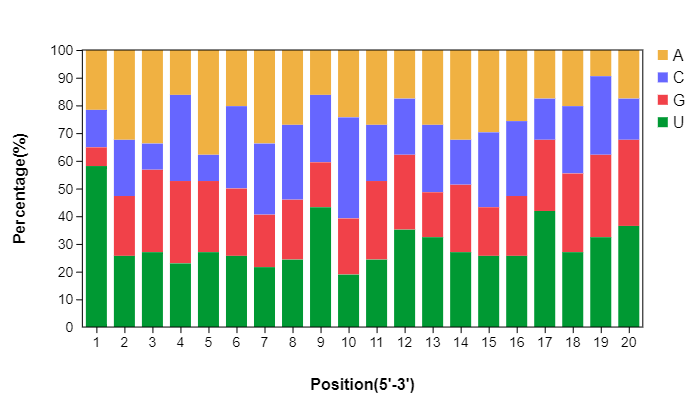

Supplement: Supplementary file 1 [file insects-12-00749-s001.zip › Supplementary Files/Figure S2.tif]

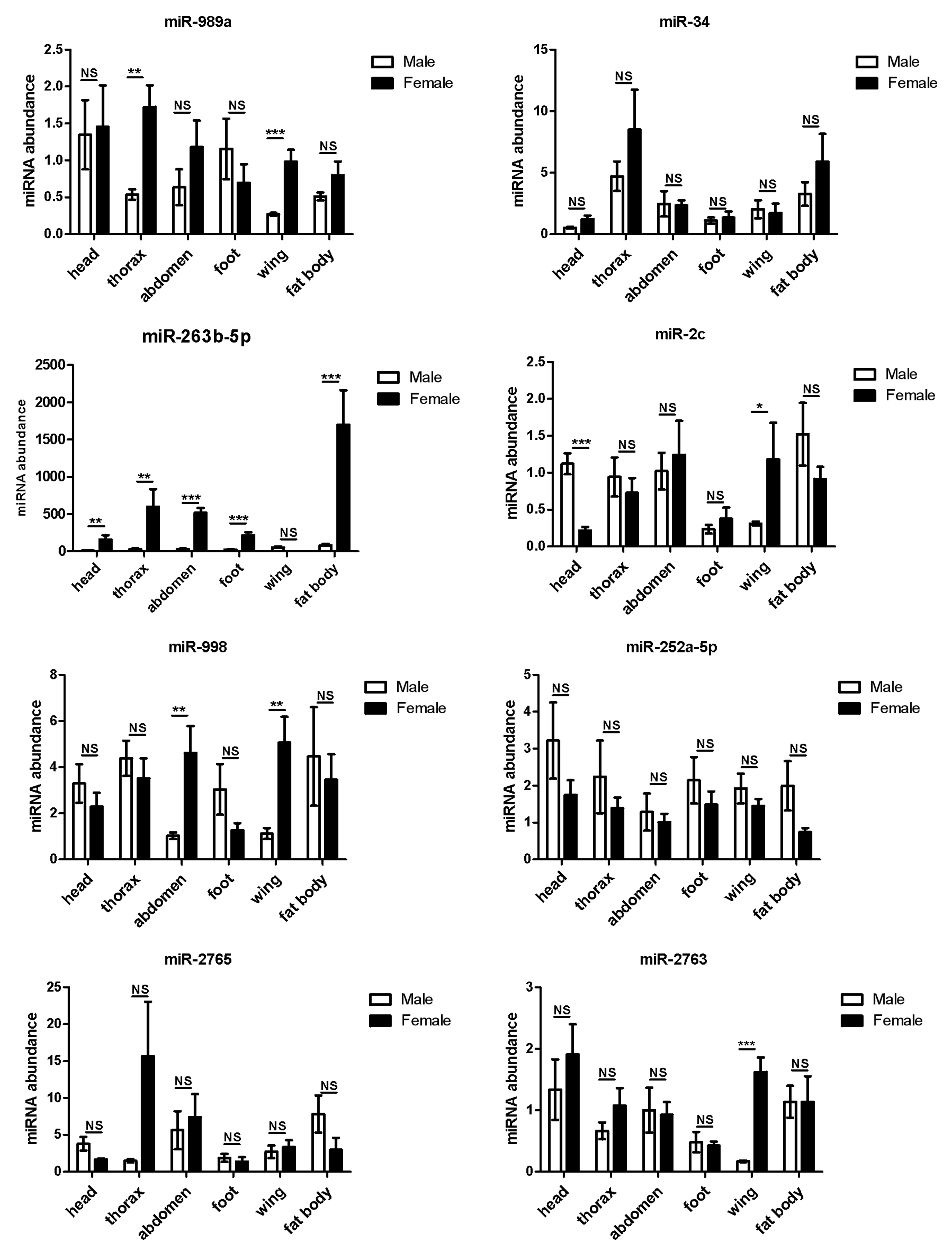

Supplement: Supplementary file 1 [file insects-12-00749-s001.zip › Supplementary Files/Figure S3.tif]
